# Increased Death of Peripheral Blood Mononuclear Cells after TLR4 Inhibition in Sepsis Is Not via TNF/TNF Receptor-Mediated Apoptotic Pathway

**DOI:** 10.1155/2021/2255017

**Published:** 2021-10-25

**Authors:** Chien-Ming Chu, Li-Chung Chiu, Chung-Chieh Yu, Li-Pang Chuang, Kuo-Chin Kao, Li-Fu Li, Huang-Pin Wu

**Affiliations:** ^1^Division of Pulmonary, Critical Care and Sleep Medicine, Chang Gung Memorial Hospital, Keelung 204, Taiwan; ^2^Department of Pulmonary and Critical Care Medicine, Chang Gung Memorial Hospital, Taoyuan 333, Taiwan and; ^3^Chang Gung University College of Medicine, Taoyuan 333, Taiwan

## Abstract

**Background:**

Apoptosis is one of the causes of immune depression in sepsis. Pyroptosis also occurs in sepsis. The toll-like receptor (TLR) 4 and receptor for advanced glycation end products (RAGE) have been shown to play important roles in apoptosis and pyroptosis. However, it is still unknown whether TLR4 inhibition decreases apoptosis in sepsis.

**Methods:**

Stimulated peripheral blood mononuclear cells (PBMCs) with or without lipopolysaccharides (LPS) and high-mobility group box 1 (HMGB1) were cultured with or without TLR4 inhibition using monoclonal antibodies from 20 patients with sepsis. Caspase-3, caspase-8, and caspase-9 activities were measured. The expression of B cell lymphoma 2 (Bcl2) and Bcl2-associated X (Bax) was measured. The cell death of PBMCs was detected using a flow cytofluorimeter.

**Results:**

After TLR4 inhibition, Bcl2 to Bax ratio elevated both in LPS and HMGB1-stimulated PBMCs. The activities of caspase-3, caspase-8, and caspase-9 did not change in LPS or HMGB1-stimulated PBMCs. The cell death of LPS and HMGB1-stimulated CD8 lymphocytes and monocytes increased after TLR4 inhibition. The cell death of CD4 lymphocytes was unchanged.

**Conclusion:**

The apoptosis did not decrease, while TLR4 was inhibited. After TLR4 inhibition, there was an unknown mechanism to keep cell death in stimulated PBMCs in patients with sepsis.

## 1. Introduction

In sepsis, immune system failure and sepsis-induced immunosuppression may result in death [1]. One of the causes for this is the increased apoptosis in immune cells in sepsis [2–4]. In fact, another regulated form of cell death, pyroptosis, may occur during sepsis. Most of the time, apoptosis and pyroptosis develop concurrently in sepsis [5].

Apoptosis is the process of regulated cell death. The pathway of toll-like receptor (TLR) 4 and nuclear factor kappa-light-chain-enhancer of activated B cells (NF-*κ*B) is considered the primary signal transduction pathway involved in apoptosis [6]. The signals originate from either extracellular (extrinsic) or intracellular (intrinsic) pathways. The major marker and the route of activation of the extrinsic pathway are tumor necrosis factor (TNF) and its receptor (TNFR). Activation of the intrinsic pathway is due to mitochondrial dysfunction, with decreased expression of B cell lymphoma 2 (Bcl2) and an increase in Bcl2-associated X (Bax). The extrinsic and intrinsic pathways activate caspase-8 and caspase-9, respectively. Caspase-3 interacts with caspase-8 and caspase-9 and directly results in apoptosis after activation.

The multiligand receptor for advanced glycation end products (RAGE) is regarded as a prototypical receptor of damage-associated molecular patterns. RAGE could regulate lung fluid balance in infection-related acute respiratory distress syndrome [7]. The underlying mechanisms may involve the downregulation of both the ion channel and tight junction proteins. Through RAGE, cell pyroptosis is induced [8]. Also, pyroptosis can be prevented by RAGE deficiency in endotoxemia and bacterial sepsis [9]. RAGE plays an important role in host defense during infection.

Thus, it is reasonable to hypothesize that TLR4 or RAGE inhibition might prevent cell death in sepsis. Due to no available human monoclonal antibody to block RAGE at present time, this *in vitro* human study was designed to determine the role of TLR4 inhibition in apoptosis in patients with sepsis. Lipopolysaccharides (LPS) and high-mobility group box 1 (HMGB1) were used as ligands of TLR4 and RAGE [10, 11]. Improving the understanding of the mechanisms underlying cell death may lead to the development of novel therapeutic strategies for patients with sepsis.

## 2. Materials and Methods

### 2.1. Participants and Definitions

From August 2018 to July 2019, 20 patients who were admitted to a 20-bed intensive care unit (ICU) in a regional teaching referral hospital for sepsis were enrolled in this study.

Sepsis was defined as a suspected or documented infection with an acute increase (≥2) in the Sequential Organ Failure Assessment score. Septic shock was defined as sepsis with a blood lactate level > 18 mg/dL and hypotension that was unresponsive to fluid resuscitation and required vasopressors to maintain mean arterial pressure ≥ 65 mmHg during the first three days following ICU admission. Respiratory failure was defined as ventilation dysfunction requiring invasive ventilator support. Acute renal failure is defined as a rapid increase in creatinine level (>0.5 mg/dL), urine output < 0.3 mL/kg/h over 24 h, or anuria over 12 h [12]. Jaundice was defined as hyperbilirubinemia (total bilirubin > 2 mg/dL), whereas thrombocytopenia was defined as a platelet count < 150,000/*μ*L. Disease severity was assessed based on the Acute Physiology and Chronic Health Evaluation (APACHE) II score [13].

Standard treatment according to guidelines was provided to all patients [14]. Patients who survived longer than 28 days after ICU admission were defined as survivors. All comorbidities and past histories were recorded. The patients' close family members provided informed consent for the collection of samples and subsequent analysis. All methods were carried out in accordance with relevant guidelines and regulations. The Institutional Review Board at Chang Gung Memorial Hospital approved this study (103-7093B, 104-8013C).

### 2.2. Peripheral Blood Mononuclear Cell (PBMC) Preparation

Whole blood (20 mL) was obtained from each patient at 08:30 AM within 48 hours of admission to the ICU and immediately mixed with heparin. PBMCs were isolated via differential centrifugation over Ficoll-Paque (Amersham Biosciences, Uppsala, Sweden) within 2 h of collection.

### 2.3. Cell Culture

Every 4 × 10^6^ PBMCs were plated in three wells of a flat-bottomed 24-well plate (Nunclon, Aarhus, Denmark) in 1 mL sterile RPMI 1640 tissue culture medium containing 5% heat-inactivated bovine serum and 1 mM sodium pyruvate (Gibco, New York, USA). The cells in the 1^st^ well were not stimulated or treated. The cells in the 2^nd^ well were stimulated with 1 *μ*g/mL LPS (Sigma, Missouri, USA) [15]. The cells in the 3^rd^ well were stimulated with 1 *μ*g/mL LPS (Sigma, Missouri, USA) and treated with 1 *μ*g/mL of neutralizing monoclonal anti-TLR4 antibody (eBioscience, California, USA) [16]. The cells in the 4^th^ well were stimulated with 200 ng/mL recombinant human (rh) HMGB1 (Sigma, Missouri, USA) [17]. The cells in the 5^th^ well were stimulated with 200 ng/mL rhHMGB1 (Sigma, Missouri, USA) and treated with 1 *μ*g/mL of neutralizing monoclonal anti-TLR4 antibody (eBioscience, California, USA). The plate was incubated at 37°C in 5% CO_2_ for 48 h. Five hundred *μ*L of 0.05% trypsin was used to resuspend cells adherent to the cell culture well wall. The cells were used for cell analysis.

### 2.4. Enzymatic Caspase-3, Caspase-8, and Caspase-9 Assay

Caspase-3, caspase-8, and caspase-9 activities were tested according to the instructions of the caspase colorimetric assay kit (Biovision, CA, USA). PBMCs (1 × 10^6^) were lysed and centrifuged at 12000 g for 10 min. Thirty *μ*g of protein from the extracts was incubated with 100 *μ*L of enzyme-specific colorimetric substrates at 37°C for 2 h. The colorimetric release of p-nitroaniline from the substrate was measured using a 405 nm light wave. Absorbance was treated as a marker of activity.

### 2.5. Western Blot

**Figure 1:**
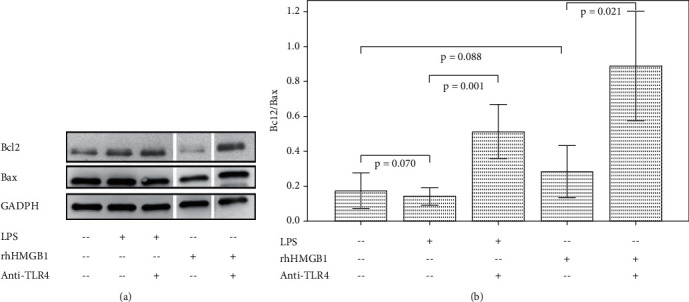
Western blot analysis of Bcl2, Bax, and GADPH levels in cultured PBMCs with/without LPS, rhHMGB1, and anti-TLR4 antibody treatment. The bar charts with one standard error show the ratio of Bcl2 to Bax (Bcl2 = B cell lymphoma 2; Bax = Bcl2-associated X; GADPH = glyceraldehyde-3-phosphate dehydrogenase; PBMCs = peripheral blood mononuclear cells; LPS = lipopolysaccharide; rhHMGB1 = recombinant human high-mobility group box 1; TLR = toll-like receptor).

Thirty *μ*g of protein from the extracts was loaded per lane. The proteins were separated using sodium dodecylsulphate polyacrylamide gel electrophoresis and transferred to nitrocellulose membranes. Membranes were blocked with 5% fat-free dry milk in 1 X Tris-buffered saline containing 0.05% Tween-20 (Sigma, Missouri, USA) for 16 h at 4°C. The blots were probed with primary antibodies against Bax (Becton Dickinson, CA, USA) (1 : 1000 dilution), Bcl-2 (Becton Dickinson, CA, USA) (1 : 1000 dilution), and glyceraldehyde-3-phosphate dehydrogenase (Becton Dickinson, CA, USA) (1 : 1000 dilution) for 1 h and then incubated with horseradish peroxidase-labeled secondary antibody. Protein bands were detected and quantified using an image analyzer (Scientific Imaging Systems V.3.6.3., Kodak Company, Stamford, CT, USA) (Figure 1(a)).

### 2.6. Flow Cytometric Analysis

**Figure 2:**
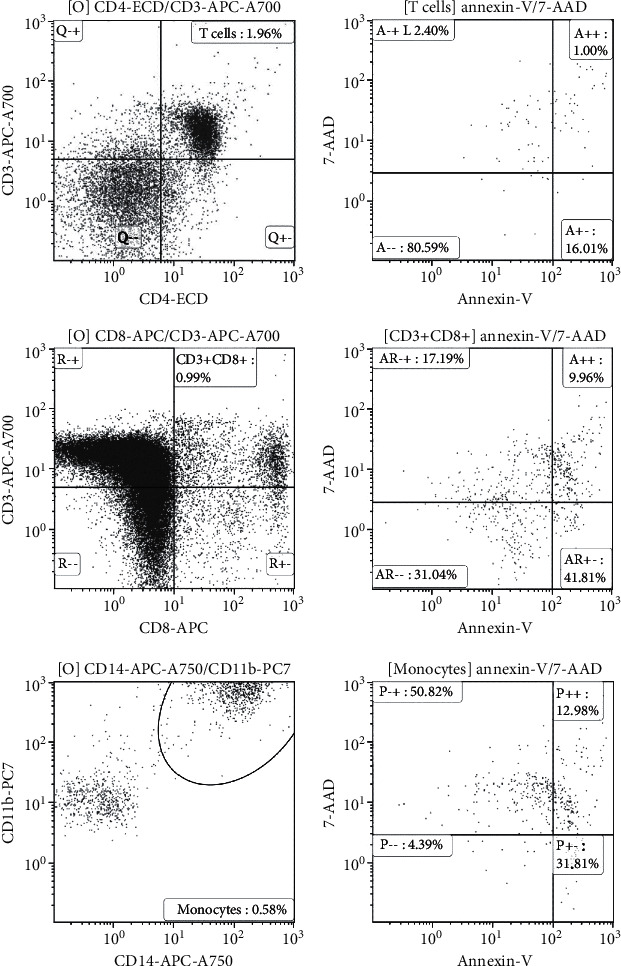
CD4 lymphocytes were identified with positive CD3 and CD4. The percentage of CD4 lymphocytes in stimulated peripheral blood mononuclear cells (PBMCs) with recombinant human high-mobility group box 1 (rhHMGB1) in a patient was 1.96%. Cell death was identified with positive 7-aminoactinomycin D (AAD) and annexin V. The percentage of cell death in CD4 lymphocytes was 1.00%. CD8 lymphocytes were identified with positive CD3 and CD8. The percentage of CD8 lymphocytes in rhHMGB1-stimulated PBMCs in a patient was 0.99%. The percentage of cell death in CD8 lymphocytes was 9.96%. Monocytes were identified with positive CD11b and CD14. The percentage of monocytes in rhHMGB1-stimulated PBMCs in a patient was 0.58%. The percentages of cell death in monocytes were 12.98%.

The residual PBMCs were suspended in 50 *μ*L of PBS and incubated in the dark for 15 min at room temperature with 10 *μ*L of CD4_ECD_, annexin V_PE_, 7-aminoactinomycin D (AAD), CD11b_PC7_, CD8_APC_, CD3_Alexa Fluor 700_, and CD14_APC-750_ antibodies. The cells were resuspended in 500 *μ*L PBS. The cell death of monocytes and lymphocytes was detected using an eight-color flow cytofluorimeter (Beckman Coulter, CA, USA). CD4 lymphocytes were identified with positive CD3 and CD4 (Figure 2). CD8 lymphocytes were identified with positive CD3 and CD8. Monocytes were identified with positive CD11b and CD14. Cell death was identified with positive 7-AAD and annexin V in gated cells.

### 2.7. Statistical Analysis

Statistical analysis was performed using Statistical Package for the Social Sciences (SPSS) software V17.0 for Windows (SPSS Inc., Illinois, USA). Differences in continuous variables in the same subjects were analyzed using the Wilcoxon signed-rank test. *p* < 0.05 was considered to indicate statistical significance.

## 3. Results

Of the 20 enrolled patients with sepsis, 14 survived for 28 days, and 6 died. Their clinical characteristics are shown in Table 1. The mean age was 69.3 years, 60% of patients were male, and the mean APACHE II score was 21.7. Moreover, 40% and 35% of patients had histories of hypertension and diabetes, respectively. The most common form of infection was pneumonia, and all patients had acute respiratory failure. Other frequent adverse events included shock, acute renal failure, and bacteremia.

### 3.1. Effect of LPS and HMGB1 Stimulation on Caspases, Bcl2/Bax, and Cell Death in Cultured PBMCs

**Figure 3:**
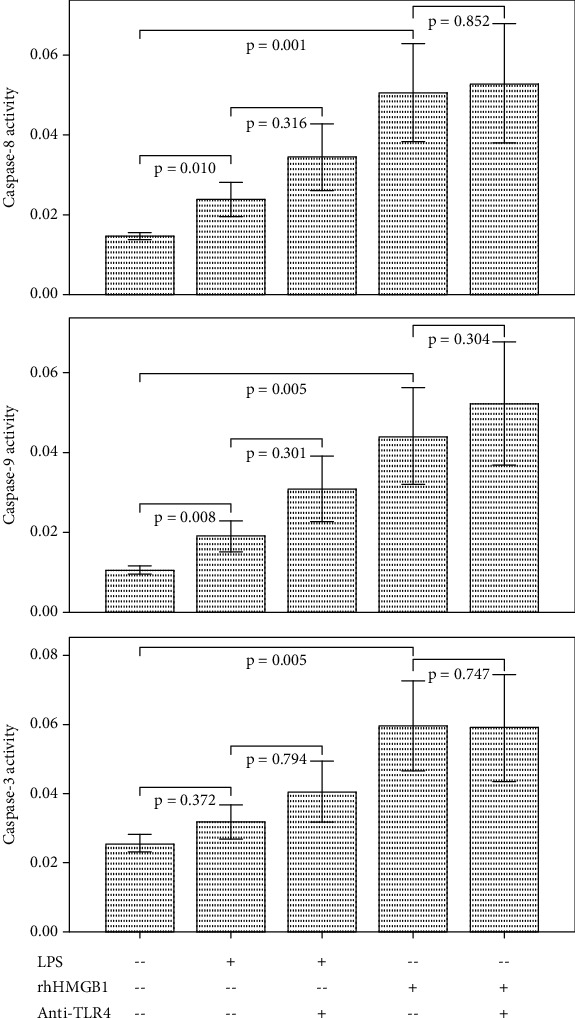
The bar charts with one standard error show caspase-3, caspase-8, and caspase-9 activities in cultured PBMCs with/without LPS, rhHMGB1, and anti-TLR4 antibody treatment (PBMCs = peripheral blood mononuclear cells; LPS = lipopolysaccharide; rhHMGB1 = recombinant human high-mobility group box 1; TLR = toll-like receptor).

**Figure 4:**
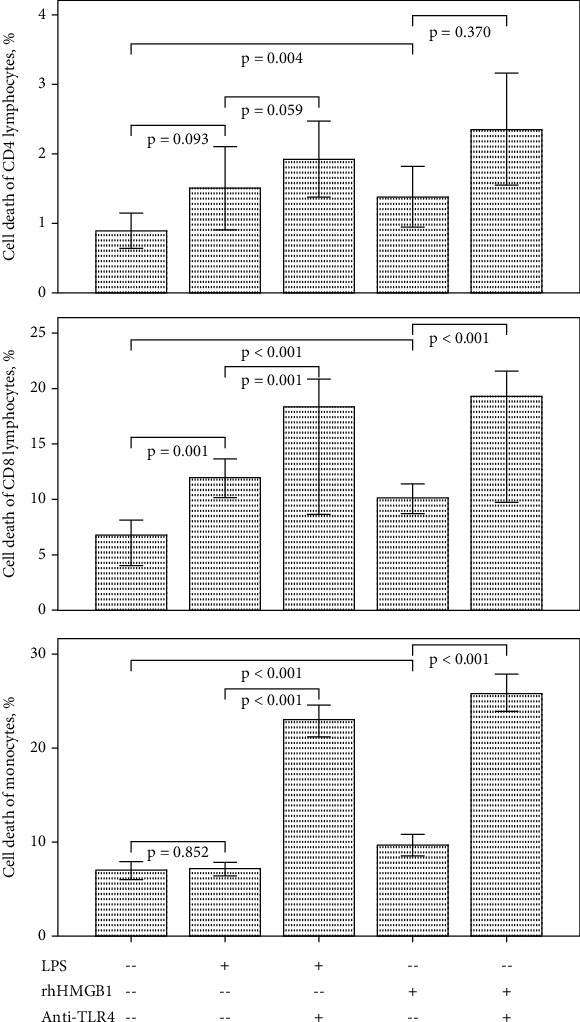
The bar charts with one standard error show cell death percentage of cultured CD4/CD8 lymphocytes and monocytes with/without LPS, rhHMGB1, and anti-TLR4 antibody treatment (LPS = lipopolysaccharide; rhHMGB1 = recombinant human high-mobility group box 1; TLR = toll-like receptor).

LPS and HMGB1 stimulation did not change the ratio of Bcl2 to Bax (Figure 1(b)). LPS and HMGB1 stimulation increased the activities of caspase-8 and caspase-9 in cultured PBMCs (Figure 3). Caspase-3 activity did not change after LPS stimulation but increased after HMBG1 stimulation. LPS stimulation increased the cell death of CD8 lymphocytes, but the cell death of CD4 lymphocytes and monocytes did not change (Figure 4). HMGB1 stimulation significantly increased the cell death of monocytes and CD4 and CD8 lymphocytes.

### 3.2. Effect of TLR4 Inhibition on Caspases, Bcl2/Bax, and Cell Apoptosis in Stimulated PBMCs

After TLR4 inhibition, Bcl2 to Bax ratio elevated both in LPS and HMGB1-stimulated PBMCs (Figure 1(b)). The activities of caspase-3, caspase-8, and caspase-9 did not change in LPS or HMGB1-stimulated PBMCs (Figure 3). The cell death of LPS and HMGB1-stimulated CD8 lymphocytes and monocytes increased after TLR4 inhibition (Figure 4). The cell death of CD4 lymphocytes was unchanged.

## 4. Discussion

**Figure 5:**
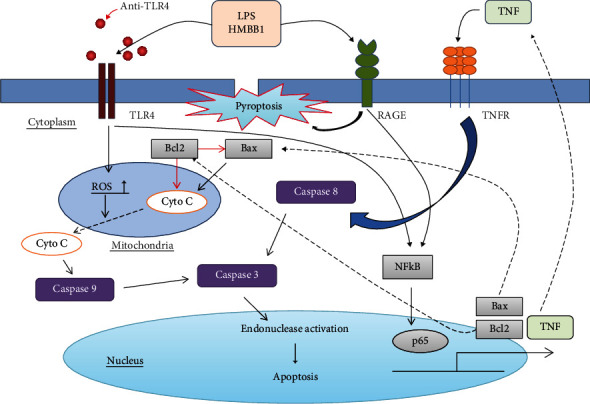
Proposed schematic mechanism of modulation of cell death. TLR4 signaling may modulate cell apoptosis related to the Bcl2 to Bax ratio in patients with sepsis. TLR4 inhibition using anti-TLR4 antibody might lead to an increase in Bcl2 to Bax ratio, which decreases Cyto C release from mitochondria. Activities of caspase-3, caspase-8, and caspase-9 did not change. Enhanced stimulation of RAGE might elevate pyroptosis, which increased cell death totally (TLR = toll-like receptor; Bcl2 = B cell lymphoma 2; Bax = Bcl2-associated X; Cyto C = cytochrome C; RAGE = receptor for advanced glycation end products; LPS = lipopolysaccharide; HMGB1 = high-mobility group box 1; TNF = tumor necrosis factor; TNFR = TNF receptor; ROS = reactive oxygen species; NF-*κ*B = nuclear factor kappa-light-chain-enhancer of activated B cells).

Our study is the first to demonstrate that TLR4 inhibition increased the cell death of stimulated PBMCs in patients with sepsis and this increased cell death was not via TNF/TNF receptor-mediated apoptotic pathway. After inhibition of TLR4 in stimulated PBMCs, activities of caspase-3, caspase-8, and caspase-9 did not change. In contrast, the ratio of Bcl2 to Bax increased after TLR4 inhibition. By elevating the ratio of antiapoptotic proteins to proapoptotic proteins, the release of cytochrome C from the mitochondria to the cytosol and initiation of the apoptotic cascade might be decreased. This would lead to a decrease in the apoptosis of stimulated PBMCs. In this study, we did not observe increased cell death of CD4 lymphocytes after TLR4 inhibition. This indicates that the suspected pathway of TLR4 in cell death (Figure 5) may be specific to some immune cells, but not CD4 lymphocytes. From a therapeutic perspective, the cell death of CD4 lymphocytes may not be modulated by TLR4 inhibition in patients with sepsis.

In this study, the cell death of stimulated CD8 lymphocytes and monocytes increased with no change in caspase-3, caspase-8, and caspase-9 activities after TLR4 inhibition. This indicates that the increase in cell death of stimulated PBMCs may not be via a caspase-3-dependent pathway in patients with sepsis. Lu et al. found that prostate cancer cells were treated with a pan-caspase inhibitor, but this treatment did not significantly affect Ophiopogonin D′-induced apoptosis [18]. This implied that Ophiopogonin D′ inhibited prostate cancer cell growth by inducing apoptosis via a caspase-independent pathway. A similar result was shown in Wu et al.'s study, which demonstrated that natural killer cell-derived extracellular vesicles were able to induce caspase-independent programmed cell death in neuroblastoma or leukemia cells [19]. These findings show that loss of cell viability caused by apoptosis can still occur without caspase-3-induced chromatin condensation, DNA fragmentation, and membrane blebbing [20]. These findings raise a fundamental question. Is caspase-3 indispensable for apoptosis in certain cell types, or is it required only because other pathways are not expressed or activated? More studies are needed to distinguish between these possibilities since rare investigators were able to detect all possible pathways in various cell types.

Apoptosis and pyroptosis cannot be clearly distinguished based on double positivity for annexin V and 7-AAD in flow cytometry because both translocation of phosphatidylserine to the outer surface of the plasma membrane and DNA fragmentation can exist in apoptosis and pyroptosis [21, 22]. Pyroptosis is a lytic cell death that occurs during inflammation mediated by inflammatory caspase-1. Active caspase-1 can activate caspase-3 to induce apoptosis, and caspase-3 also cleaves gasdermin D (GSDMD) to suppress pyroptosis during apoptosis [23, 24]. Briefly, caspase-1 activation leads to pyroptosis in GSDMD-sufficient cells, and apoptosis in GSDMD-deficient cells. The percentage of PBMC pyroptosis in sepsis and the clinical significance of pyroptosis remain unclear. By labeling caspase-1 and caspase-3 in dying cells using flow cytometry, Wang et al. found that the mean proportion of apoptotic and pyroptotic PBMCs in trauma patients with sepsis was approximately 11.5% and 12.8%, respectively [25]. This study also demonstrated that increased pyroptosis in PBMCs was associated with the development of sepsis and suggested that PBMC pyroptosis plays a role in the pathogenesis of inflammation following trauma.

In this study, the reason for the lack of increase in cell death in stimulated CD4 lymphocytes after TLR4 inhibition was unclear. One of the possibilities might be that cell death in CD4 lymphocytes in patients with sepsis was mainly via apoptosis since TLR4 inhibition could elevate the Bcl2 to Bax ratio to suppress apoptosis. And cell death in CD8 lymphocytes and monocytes might be mainly via pyroptosis or non-TNF/TNF receptor-mediated apoptotic pathways. This could also explain why TLR4 inhibition did not influence the activities of caspase-3, caspase-8, and caspase-9 in stimulated PBMCs since cell death in PBMCs might be caused through relatively enhanced stimulation of RAGE, which resulted in increased pyroptosis. More studies are necessary to elucidate the roles of apoptosis and pyroptosis in sepsis and the interaction between them.

This study has two limitations. First, the study did not investigate all molecular pathways of apoptosis and pyroptosis at the same time. The effect of TLR4 inhibition on the molecular signaling pathways of pyroptosis is still unknown. Second, the study did not perform experiments of different cell types through cell sorting by flow cytometry. This will answer the correlation of apoptosis and pyroptosis with different immune cells.

## 5. Conclusions

TLR4 plays a role in LPS and HMGB1-induced apoptosis in PBMCs of patients with sepsis. The apoptosis did not decrease, while TLR4 was inhibited using a monoclonal antibody. After TLR4 inhibition, pyroptosis may become an important type of cell death in stimulated PBMCs in patients with sepsis. Furthermore, only the TNF/TNF receptor-mediated apoptotic pathway has been studied. Therefore, it is necessary to study all apoptotic pathways. A better understanding of the mechanisms that regulate sepsis-associated cell death including apoptosis and pyroptosis will provide insights into inflammatory biology and new molecular targets for treating sepsis.

## Figures and Tables

**Table 1:** Clinical characteristics in patients with sepsis (number, mean ± standard error mean).

	All patients (*n* = 20)
Age (years)	69.3 ± 3.5
Male (%)	12 (60.0)
APACHE II score	21.7 ± 1.5
History (%)	
Chronic obstructive pulmonary disease	2 (10.0)
Heart failure	2 (10.0)
Hypertension	8 (40.0)
Diabetes mellitus	7 (35.0)
Old cerebral vascular accident	3 (15.0)
End-stage renal disease	3 (15.0)
Liver cirrhosis	3 (15.0)
Infection source	
Pneumonia	14 (70.0)
Urinary tract infection	5 (25.0)
Others	1 (5.0)
Adverse event	
Acute respiratory failure	20 (100.0)
Gastrointestinal bleeding	1 (5.0)
Acute renal failure	9 (45.0)
Shock	11 (55.0)
Thrombocytopenia	6 (30.0)
Jaundice	3 (15.0)
Bacteremia	8 (40.0)
28-day mortality	6 (30.0)

Abbreviations: APACHE = Acute Physiology and Chronic Health Evaluation.

## Data Availability

The datasets used and/or analyzed during the current study are available from the corresponding author upon reasonable request.
